# Supplementary material for: Identification of photoperiod-induced specific miRNAs in the adrenal glands of Sunite sheep (Ovis aries)
Source: Front Vet Sci. 2022 Jul 22;9:888207. doi: 10.3389/fvets.2022.888207 (PMC9354845; doi:10.3389/fvets.2022.888207)
Supplement: Supplementary file 1 [file Data_Sheet_1.ZIP › Supplementary file/miRDeep2 file.zip!/miRDeep2 file/Novel_Structure/LP42A1/Novel_312.pdf]

Provisional ID : NC_019459.2_5575
Score total : 1.9
Score for star read(s) : -1.3
Score for read counts : 0
Score for mfe : 1.6
Score for randfold : 1.6
Score for cons. seed :
Total read count : 118
Mature read count : 118
Loop read count : 0
Star read count : 0

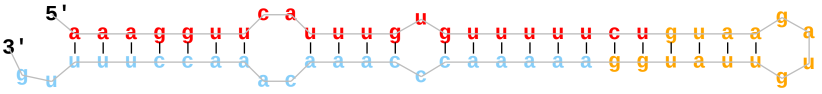

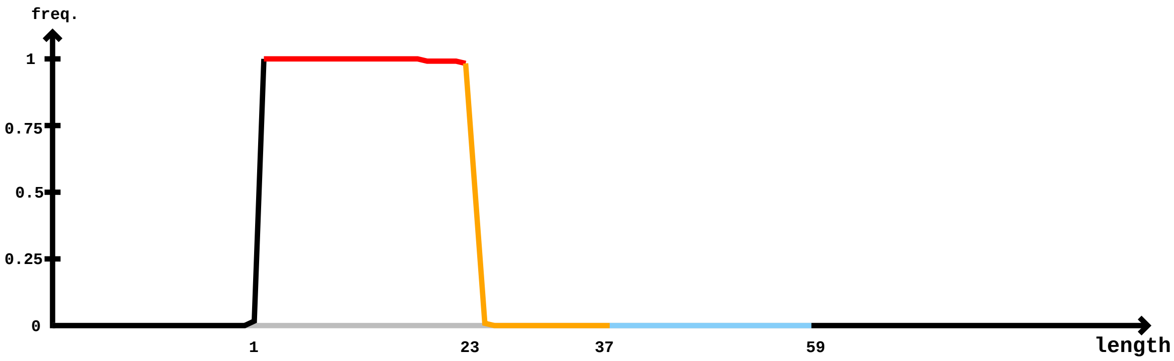

Mature Star

```
5'- uggaaauacuagauuggccaaaagguucauuuguguuuuucuguaagauguuaugggaaaaacccaaacaaaccuuuuggccaacccaauauaaaacuacucuguauaagucu   -3'   exp
    .....((((.(((((((((((((((((..((((.((((((((((((....)))))))))))).))))..))))))))))))))...............)))))))......    reads   mm   sample
    ...................aaaagguucauuuguguuuuuc.......................................................................    1       0    seq
    ...................aaaagguucauuuguguuuuucu......................................................................    1       0    seq
    ....................aaagguucauuuguguu...........................................................................    1       0    seq
    ....................aaagguucauuuguguuuuucu......................................................................    114     0    seq
    ....................aaagguucauuuguguuuuucugu....................................................................    1       0    seq
```
